# Successful Pregnancy in a Couple with Severe Male Factor Infertility after Selection of Sperm with Cytoplasmic Droplets

**DOI:** 10.1155/2015/276931

**Published:** 2015-07-26

**Authors:** Jenna Bellish, David H. McCulloh, Khaliq Ahmad, Peter G. McGovern

**Affiliations:** ^1^Department of Obstetrics and Gynecology, Rutgers-New Jersey Medical School, 185 South Orange Avenue, MSB-E506, Newark, NJ 07103, USA; ^2^University Reproductive Associates, 214 Terrace Avenue, Hasbrouck Heights, NJ 07604, USA; ^3^New York University Langone Medical Center, NYU Fertility Center, 660 1st Avenue, New York, NY 10016, USA; ^4^Department of Obstetrics and Gynecology, Texas Tech University School of Medicine, Lubbock, TX 79430, USA; ^5^Department of Obstetrics and Gynecology, Mount Sinai St. Luke's Roosevelt Hospital, 1000 Tenth Avenue, Suite 10C, New York, NY 10019, USA

## Abstract

We present live births resulting from two separate IVF cycles in a couple in which ICSI was performed with sperm specifically selected for presence of small cytoplasmic droplets. These cycles followed previous cycles using standard sperm selection methods in which very poor embryo development and no pregnancies ensued. The male partner was diagnosed with severe male factor infertility including elevated DNA fragmentation.

## 1. Introduction

Male factor infertility is responsible for approximately 40–50% of fertility problems worldwide. While it is largely believed that poor sperm function affects the ability of sperm to fertilize oocytes, sperm pathology may affect fertility after fertilization and become obvious as poor embryo morphology or development or may go altogether unnoticed in the embryology laboratory. DNA damage in sperm has been associated with poor pregnancy outcome but is not necessarily associated with poor fertilization. Few treatments have been suggested for sperm with a high degree of DNA damage.

Cytoplasmic droplets on sperm have been statistically correlated with the elevated levels of reactive oxygen species in sperm samples although direct evidence of their role in infertility is not well substantiated [1–3]. Small cytoplasmic droplets on sperm in the ejaculate may indicate that the sperm was more recently released from its surrounding Sertoli cells and may have been subjected to DNA damage for a briefer time.

In this report, we present live births resulting from in vitro fertilization (IVF) in which intracytoplasmic sperm injection (ICSI) was performed with ejaculated sperm selected for presence of small cytoplasmic droplets when the male partner was diagnosed with severe male factor infertility including DNA fragmentation.

A couple was referred to our center for fertility evaluation.

*History and Diagnosis of the Female Partner*. The female partner was a 30-year-old nullipara with a greater than one year of primary infertility. She was 67 inches tall and weighed 212 pounds (BMI = 33). She had a past history significant for laparoscopic removal of bilateral dermoid cysts (right was 5 cm and left was 2 cm) in 1995 and a laparoscopic cholecystectomy in 2003. She also reported a history of treatment for chlamydia in 1997. The remainder of the history was noncontributory and physical exam was within normal limits.

Evaluation included a normal hysterosalpingogram and normal laboratory testing. All testing showed normal ovarian function, with a day 3 FSH level of 8 IU/L and antral follicle count of 15. She responded well to gonadotropins, and the dose actually needed to be reduced in her second IVF cycle as her follicular response was exuberant.

*History and Diagnosis of the Male Partner*. The male partner was 34 years old, 70 inches tall, and weighing 252 pounds (BMI = 36.1), who had a history of 2 childhood surgeries for cryptorchid testes, with no other significant history. Semen analyses revealed severe male factor infertility (oligoteratozoospermia) with sperm counts between 0.14 and 4.8 million sperm/mL. Sperm motility varied widely from 20 to 80%. The total number of motile sperm per ejaculate ranged from 0.06 to 4.8 million motile sperm. Morphology determinations assessed by Kruger's strict criteria on two occasions were low (3% and 4%). Physical examination by a urologist and hormonal panel (FSH, LH, testosterone, TSH, and prolactin) was normal. Chromosome studies and Y-chromosome microdeletion testing were performed on the male partner, and both tests were normal. The male partner did not receive any therapy throughout the entire course of the couple's treatment. 

*Treatment of the Couple*. The patients (female patient age = 31 years) underwent their first cycle of IVF with ICSI. The controlled ovarian hyperstimulation (COH) involved luteal downregulation with day 21 leuprolide acetate followed by daily gonadotropin injections beginning after the onset of menses. A total of 3225 IU of recombinant FSH (Gonal F, Serono Pharmaceuticals) was administered prior to triggering on day 10 with 10,000 IU of human chorionic gonadotropin (hCG). Endometrial thickness was 12 mm at trigger. A total of 14 eggs were retrieved via transvaginal, ultrasound guided follicular aspiration. Semen characteristics were Volume 3.0 mL; Count = 1.6 M/mL; Motility = 27%. Following ICSI, eight oocytes were fertilized. Two poor quality blastocysts (both B/C) were transferred. None of the remaining embryos were cryopreserved due to poor quality. Luteal support was provided with intramuscular injections of Progesterone in oil 50 mg daily. Pregnancy did not occur.

In the couple's second cycle (female patient age = 31 years), COH involved luteal downregulation with day 21 leuprolide acetate followed by daily gonadotropin injections beginning after the onset of menses. A total of 3225 IU of recombinant FSH (Gonal F, Serono Pharmaceuticals) was administered prior to triggering on day 9 with 10,000 IU of human chorionic gonadotropin (hCG). Endometrial thickness was 13 mm at trigger. Twelve eggs were retrieved. Semen characteristics were Volume 2.3 mL; Count = 1.4 M/mL; Motility = 20%. Eleven oocytes were subjected to ICSI and 7 were fertilized. Two poor quality early blastocysts (C/C and B/C) were transferred. None of the remaining embryos were cryopreserved due to poor quality. Luteal support was provided with intramuscular injections of Progesterone in oil 50 mg daily. Pregnancy did not occur.

Following the second unsuccessful IVF/ICSI attempt, the couple faced the decision of proceeding to donor sperm insemination versus attempting another IVF cycle. At that point, our IVF team believed that sperm DNA fragmentation testing would help to determine the most appropriate course of therapy. Sperm DNA fragmentation analysis was then performed (SCSA Diagnostics, Brookings, SD). There was a 43.4% DNA Fragmentation Index (DFI) (DFI reference range: ≤15%, excellent sperm DNA integrity; ≥30%, fair to poor sperm DNA integrity) which is the % of sperm cells containing damaged DNA. This result places the patient in the group of fair to poor sperm integrity. There was also a 15.9% High DNA Stainability (HDS) (HDS reference range: ≤15%, normal; >15%, above normal) which indicates an elevated % age of sperm with immature chromatin. The testing was repeated 4 weeks later with similarly elevated results of a 33.0% DFI and a 21.1% HDS.

Sperm collected through testicular biopsy have been demonstrated to have less DNA damage than sperm found in the same man's ejaculate [4]. However, prior to performing surgery on the male, we wished to attempt an approach that was less invasive than testis biopsy. Based on the high sperm fragmentation and poor sperm integrity, we hypothesized that choosing recently matured sperm might avoid the use of sperm that had experienced prolonged exposure to endogenous agents that promote DNA fragmentation. Since sperm from this patient exhibited a high incidence of DNA fragmentation, a temporally “younger” sperm likely had less time to be exposed to the process by which DNA damage was occurring. We had noted a high percentage of sperm in testicular biopsies for other patients that had cytoplasmic droplets. Since all sperm normally have cytoplasmic droplets prior to reaching the epididymis, sperm that still had droplets retained in the ejaculate were hypothesized to be “younger.” Despite the correlational data suggesting associations with poor fertility [1–3, 5–7] others have reported that cytoplasmic droplets are normal features of sperm [8, 9]. These sperm were selected for use during ICSI with the patient's third cycle. Sperm were selected that had a small (<1/3 size of the head) droplet. The sperm were then immobilized, as is customary for ICSI by using the tip of the ICSI pipette to drag the sperm along the bottom of the dish by the tail. During this immobilization procedure, cytoplasmic droplets typically become detached, before injection into the oocyte.

In the third cycle, the male partner did not seek any therapy outside of our facility. COH (female partner's age = 31 years) involved luteal downregulation with day 21 leuprolide acetate followed by daily gonadotropin injections beginning after the onset of menses. A total of 2700 IU of recombinant FSH (Gonal F, Serono Pharmaceuticals) was administered prior to triggering on day 10 with 10,000 IU of human chorionic gonadotropin (hCG). Endometrial thickness was 14 mm at trigger. 8 oocytes were retrieved in this cycle. Semen characteristics were Volume 1.0 mL; Count = 0.14 M/mL; Motility = 40%. Three oocytes were fertilized with ICSI and were transferred at the cleavage stage of which 2 were good quality (4 cell A/B and 6 cell B) along with one poor quality embryo (7 cell C). Luteal support was provided with intramuscular injections of Progesterone in oil 50 mg daily. This transfer resulted in an uncomplicated singleton pregnancy. The female partner delivered a healthy male infant (6 lbs 12 oz) at full-term. No birth defects were noted and the child is progressing well at age 7.

Two years after the third IVF cycle (female patient age = 33 years), the couple returned when they decided to attempt another pregnancy. Repeat in vitro fertilization with ICSI was performed, again choosing sperm selected by presence of a small cytoplasmic droplet. No repeat SCSA was performed since patients with poor SCSA results seldom improve with time. COH involved luteal downregulation with day 21 leuprolide acetate followed by daily gonadotropin injections beginning after the onset of menses. A total of 5550 IU of gonadotropin comprising 4800 IU of highly purified FSH (Bravelle, Ferring Pharmaceuticals) and 750 IU of highly purified human menopausal gonadotropin (Menopur, Ferring Pharaceuticals) was administered prior to triggering on day 11 with 10,000 IU of human chorionic gonadotropin (hCG). Endometrial thickness was 9 mm at trigger. Nine oocytes were retrieved. Semen characteristics were Volume 2.1 mL; Count = 0.67 M/mL; Motility = 50%. Seven oocytes were fertilized with ICSI. Luteal support was provided with intramuscular injections of Progesterone in oil 50 mg daily. Three good quality cleavage stage embryos were transferred (two 8 cell A/B and one 7 cell A/B), resulting in 3 gestational sacs. There were noted to be 2 fetuses within one sac; however, one of those was clearly nonviable. Thus only two fetal heartbeats were ever detected. The patient delivered healthy twins at 36 weeks (female 4 lbs 15 oz and male 5 lbs and 12 oz). No birth defects were noted and the children are progressing well at age 5.

## 2. Discussion

This is the first report of a couple achieving successful pregnancy and live birth resulting from IVF with sperm specifically selected for the presence of cytoplasmic droplets. In addition, this is the first report of a technique used to rectify sperm DNA damage as assessed by the SCSA. The relationship between DNA fragmentation and fertility has been investigated, but the relationship between cytoplasmic droplets and DNA fragmentation has not. The concept of using spermatozoa with less DNA fragmentation by selecting for small cytoplasmic droplets as a marker of “younger” sperm is promising but remains unproven.

Among the tests used to assess DNA damage is the Sperm Chromatin Structure Assay (SCSA). Sperm DNA fragmentation analysis is currently recommended by some authorities in specific situations: unexplained infertility, persistent infertility after treatment of the female partner, recurrent miscarriage, exposure to reproductive toxins, male cancer before and after treatment, abnormal semen analysis, or male age >50 years [10]. A DNA fragmentation index consistently >30% is associated with poorer fertility prognosis (increased risk of spontaneous abortion, failure to achieve pregnancy, longer time to successful pregnancy, and more IVF cycles).

During the process of spermatogenesis, extensive reorganization occurs in both the nucleus and the cytoplasm. Excess cytoplasm, present as a proximal droplet on the midpiece, migrates distally and is normally extruded in the corpus and proximal cauda of the epididymis. Therefore, disappearance of this excess cytoplasm may be a marker of longevity in the male reproductive tract.

“Cytoplasmic droplet” is the term used in the literature to describe what the WHO terms an “abnormal cytoplasmic droplet” which is >1/2 [to 1/3] the size of the sperm head in an air-dried, fixed, and stained preparation. Cytoplasmic droplets less than that size, present as vesicles at the neck of the sperm, are likely normal remnant of cytoplasm on sperm produced by a normal testicle. As these smaller droplets are not considered abnormal by the WHO guidelines and therefore not noted on reports, their relation to fertility is unknown. While there is data showing that large cytoplasmic droplets are related to a variety of abnormalities [1–3, 5], including poor motility, reduced zona pellucida binding, and increased chromatin breaks and DNA damage, studies suggest that small droplets are normal structures of spermatozoa [8, 9].

There is much disparity in the literature with regard to sperm function and fertility outcomes with presence of a cytoplasmic droplet. Studies done with spermatozoa of domestic animals demonstrate that retention of a proximal droplet in the ejaculate is related to infertility. This is related to a failure of normal maturation of the sperm, as the droplet normally migrates along the midpiece distally during epididymal transport.

Human spermatozoa are different in that when the cytoplasmic droplet remains there is no migration, and the droplet remains at the neck. Studies in human spermatozoa that associate poor sperm function with the presence of a cytoplasmic droplet have used the WHO criteria and used at least 1/3 of the size of the sperm head as the defining parameter in the study. Conversely, one recent study showed that cytoplasmic droplets, defined as regular distensions at the neck or along the midpiece, are not deleterious to sperm motility in humans [7]. In distinction with small cytoplasmic droplets, large irregular material along the midpiece is termed residual cytoplasm and is believed to be a sign of poor quality sperm. Retained, extra cytoplasm on air-dried smears has been linked to sperm immaturity by immunocytochemistry and morphologic analysis [11, 12]

While the intention of avoiding DNA damage by selecting less aged sperm is intriguing, the relationship between immaturity and DNA fragmentation is unclear. Further investigation will be necessary to determine whether the selection of sperm by the presence of a small cytoplasmic droplet is an effective method of avoiding sperm with excessive DNA damage. Other methods such as performance of testicular biopsy to obtain sperm may prove to be a more effective method of obtaining sperm with minimal DNA damage [4]. However, this method described herein may provide patients with an option that does not require invasive testicular biopsy procedures.
